# Biomechanically and biochemically functional scaffold for recruitment of endogenous stem cells to promote tendon regeneration

**DOI:** 10.1038/s41536-022-00220-z

**Published:** 2022-04-26

**Authors:** Jing Cui, Liang-Ju Ning, Fei-Peng Wu, Ruo-Nan Hu, Xuan Li, Shu-Kun He, Yan-Jing Zhang, Jia-Jiao Luo, Jing-Cong Luo, Ting-Wu Qin

**Affiliations:** 1 Laboratory of Stem Cell and Tissue Engineering, Orthopedic Research Institute, State Key Laboratory of Biotherapy and Cancer Center, West China Hospital, Sichuan University and Collaborative Innovation Center of Biotherapy, Chengdu, China; 2 Department of Orthopedic Surgery, West China Hospital, Sichuan University, Chengdu, China; 3 Panzhihua Central Hospital, Panzhihua, China

**Keywords:** Stem-cell niche, Biomaterials - cells, Regeneration, Stem-cell differentiation

## Abstract

Tendon regeneration highly relies on biomechanical and biochemical cues in the repair microenvironment. Herein, we combined the decellularized bovine tendon sheet (DBTS) with extracellular matrix (ECM) from tendon-derived stem cells (TDSCs) to fabricate a biomechanically and biochemically functional scaffold (tECM-DBTS), to provide a functional and stem cell ECM-based microenvironment for tendon regeneration. Our prior study showed that DBTS was biomechanically suitable to tendon repair. In this study, the biological function of tECM-DBTS was examined in vitro, and the efficiency of the scaffold for Achilles tendon repair was evaluated using immunofluorescence staining, histological staining, stem cell tracking, biomechanical and functional analyses. It was found that tECM-DBTS increased the content of bioactive factors and had a better performance for the proliferation, migration and tenogenic differentiation of bone marrow-derived stem cells (BMSCs) than DBTS. Furthermore, our results demonstrated that tECM-DBTS promoted tendon regeneration and improved the biomechanical properties of regenerated Achilles tendons in rats by recruiting endogenous stem cells and participating in the functionalization of these stem cells. As a whole, the results of this study demonstrated that the tECM-DBTS can provide a bionic microenvironment for recruiting endogenous stem cells and facilitating in situ regeneration of tendons.

## Introduction

Tendon injuries, particularly large segmental tendon injuries, impose noticeable clinical burdens on healthcare systems around the world. It is well known that natural healing of tendons forms fibrotic scars rather than regenerative tissue due to the lack of vascularity, lack of highly differentiated tenocytes, and limited proliferation capability^1^. Therefore, the natural tendon healing process always results in poor tendon quality and inferior biomechanical properties^2^. Clinical interventions, including scaffold-mediated repair or conventional surgeries generally produce excellent results for small-to-mid-sized tendon defects^3–5^. However, for large-to-massive defects, tendon repair is facing great challenges due to a mechanically compromised microenvironment, which often leads to a re-tear rate as high as 94%^6^. Therefore, obtaining a well-performed scaffold with proper dimensions and biomechanical matching to repair large-to-massive defects is highly desirable.

The biomechanical function of a scaffold for tendon repair is known to play important roles in influencing cell fate and tendon regeneration^7–9^. This biomechanical function may include three aspects: the stress-strain characteristics of the intact scaffold, the scaffold surface stiffness, and the suture retention strength of the scaffold. The ideal stress-strain characteristics of the scaffold should match the native tendon^10^. Simultaneously, the surface stiffness of the scaffold could affect the fate of cell differentiation^7^. Studies have confirmed that soft matrices are neurogenic, moderate rigid matrices are tenogenic, and comparatively rigid matrices prove osteogenic^11,12^. Additionally, in tendon repair, the scaffold needs an appropriate suture and fixation with the host tendon to transmit muscular contraction force^13^. The suture retention strength of the scaffold has proven to be critical for tendon reconstruction surgery, as it determines the scaffold resistance to suture pull-out^14^. Actually, the stress-strain characteristics and suture retention capacity of the scaffold ensure the transmission of tensile stress. The tensile stress stimulation of the scaffold affects the cell tenogenic differentiation^15^. A previous study demonstrated that tensile mechanical stimulation of BMSC-DTSs (BMSC seeded on decellularized tendon slices) constructs upregulated the expression of tendon-related genes and promoted cell tenogenic differentiation^16^.

In addition to biomechanical function, biochemical function of a scaffold is also important factors in the microenvironment of tendon repair and regeneration. Recent advances of in situ tissue engineering may offer a promising treatment for tendon repair^17^. It focuses on the inductive properties of bioactive factors of the scaffold to promote the migration of autologous cells to the site of injury and to direct differentiation in vivo, thereby completing tissue repair and regeneration^17^. Studies have demonstrated that the addition of SDF-1 to scaffolds improves the recruitment of stem cells^18,19^. In addition, Kim et al. proved that the ability of nanofibrous scaffolds to recruit autologous stem cells can be improved by combining bioactive peptides^20^. The roles of bioactive factors have been well characterized during tendon healing. The addition of TGF-β1 to the scaffold led to a statistically significant increase in the amount of collagen I and III produced^21^. Chen et al. showed that BMSCs can secrete SDF-1, VEGF, IGF-1, and PDGF-1 to recruit macrophages and enhance wound healing^22^. TGF-β can improve the mechanical strength of repaired Achilles tendons by modulating collagen synthesis, cross-link formation, and matrix remodeling^23^.

Recently, the application of the cell-derived ECM has been a growing interest because it facilitates the regeneration of specific tissues^24–27^. The ECM contains proteoglycan and bioactive factors that play a critical role in modulating the fate of stem cells^28,29^. Previous studies have shown that biglycan (BGN) and fibromodulin (FMOD), as important components of the extracellular matrix of tendons, determine the tenogenic differentiation of tendon-derived stem cells (TDSCs)^30^. Moreover, a number of authors have successfully prepared scaffolds that were coated with cell-secreted ECM, and these composite scaffolds can induce stem cells migration in vitro^31^ and promote tendon regeneration in vivo^32^.

The decellularized tendon extracellular matrix scaffold, as a tissue-specific scaffold, has great potential for tendon repair/reconstruction^33–35^. The advantages of natural ECM scaffolds are their well-matched biomechanical properties and the appropriate biochemical and structural composition to guide cell growth^36^. Promisingly, in our previous study, we have successfully prepared a decellularized bovine tendon sheet (DBTS) with proper dimensions and adequate availability^37^. Fortunately, the DBTS scaffold exhibit matching biomechanical characteristic and excellent suture retention strength, which provide a mechanical microenvironment for tendon repair^38^. Zhang et al. further demonstrated the ability of DBTS to remodel and integrate into the host tendon in the reconstruction of Achilles tendon defects in a rabbit model^39^. However, the preparation process of the scaffold inevitably loses some bioactive factors, which would affect the biological activity of the scaffold in vitro and the efficacy of tendon regeneration in vivo. TDSCs, as tendon tissue-specific stem cells, have been commonly used in tendon tissue engineering^29,30,40^. Moreover, TDSCs have a stronger potential for tenogenic differentiation than bone marrow mesenchymal stem cells (BMSCs)^29^. Further studies confirmed that the extracellular matrix components secreted by TDSCs contain growth factors that regulated the migration of stem cells^31^, proteoglycans that determined tenogenic differentiation^30^, and anti-inflammatory cytokines that prevented immunological rejection^41^. Especially, cell-derived ECM can be deposited in scaffolds with various three-dimensional shapes and architectures, equipped with additional functionalities, and customized by selecting cells from specific tissues or individuals^42^. However, whether the DBTS scaffold coated with TDSCs-derived ECM could augment the biological activity to promote tendon regeneration has not been investigated.

The objective of this study was to combine DBTS with ECM secreted by TDSCs to fabricate a biomechanically and biochemically functional scaffold (tECM-DBTS), which not only provides good biomechanical support for tendon regeneration but also enhances biological activity by increasing the amount of bioactive factors. Therefore, we hypothesized that the tECM-DBTS scaffold has a better ability to synergistically optimize the biomechanical and biochemical microenvironment for successful Achilles tendon repair in a rat model.

## Results

### Fabrication and characterization of tECM-DBTS

**Fig. 1:**
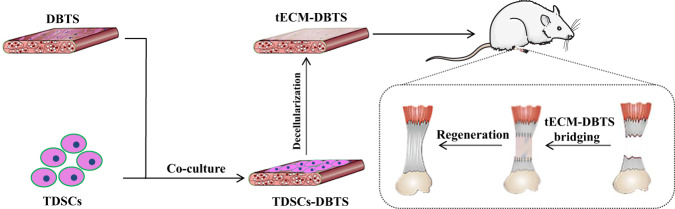
Schematic illustration of the tECM-DBTS preparation process and animal model. Abbreviations: DBTS, decellularized bovine tendon sheet; TDSCs-DBTS, TDSCs co-culture with DBTS; tECM-DBTS, DBTS scaffold combined with TDSCs-derived ECM.

**Fig. 2:**
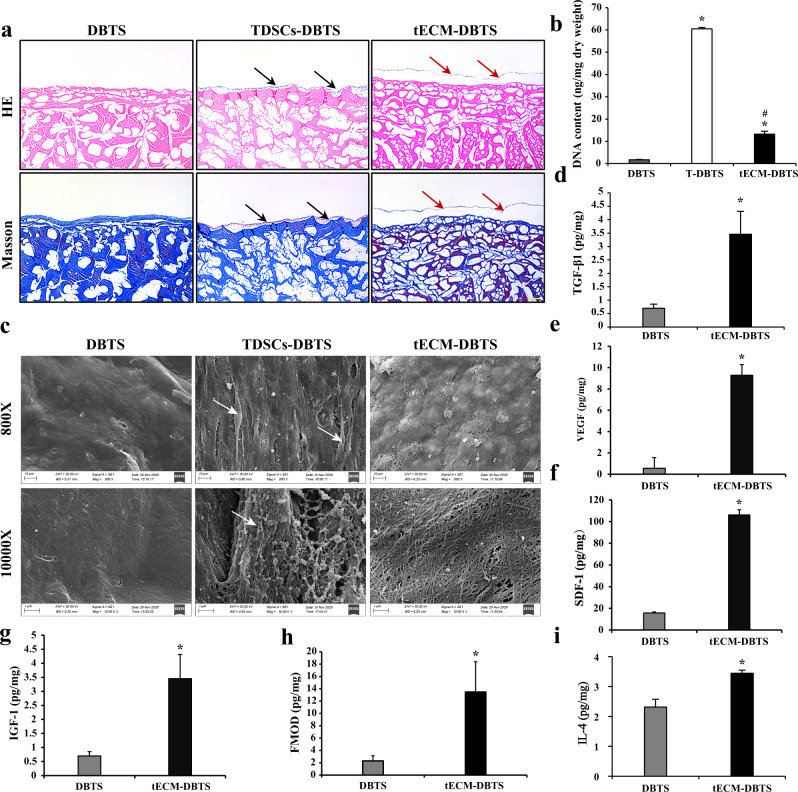
Preparation and evaluation of the tECM-DBTS scaffold. **a** HE and Masson staining of the DBTS, TDSCs-DBTS and tECM-DBTS. Black arrows: dense cell sheets produced by TDSCs; Red arrow: ECM secreted by TDSCs. **b** DNA content before and after the decellularization process as determined by the PicoGreen assay (*n* = 4), T-DBTS: TDSCs co-culture with DBTS. **c** The surface ultrastructure of the DBTS, TDSCs-DBTS and tECM-DBTS was determined by SEM. The white arrow showed that the TDSCs on the DBTS. The content of TGF-β1 (**d**), VEGF (**e**), SDF-1 (**f**), IGF-1 (**g**), FMOD (**h**) and IL-4 (**i**) in the DBTS and tECM-DBTS scaffold was determined by ELISA analysis (*n* = 4). *Signifies a *p* < 0.05 as compared to the DBTS. #Signifies a *p* < 0.05 as compared to the T-DBTS. Scale bar, a: 100 μm; b: 10 μm (800X), 1 μm (10000X).

The tECM-DBTS was prepared as illustrated in Fig. 1. H&E and Masson staining showed that no cellular components were observed in the tECM-DBTS scaffold after decellularization while preserving the epitenon. After 15 days of culture, dense cell sheets were formed by TDSCs on the epitenon of the DBTS. After decellularization, the epitenon of DBTS was completely covered by a membrane-like structure, which was the ECM secreted by TDSCs (Fig. 2a). The DNA quantification assay showed that the residual DNA content was significantly reduced in the tECM-DBTS scaffold compared to TDSCs-DBTS (Fig. 2b).

SEM images of sections revealed changes in the surface morphology before and after modification with ECM of stem cells. Before modification, the epitenon of DBTS was quite distinct in the DBTS, and after modification, the granular structure of ECM secreted by TDSCs accumulated on the surface of epitenon to form a relatively interlaced microstructure network (Fig. 2c).

The bioactive factor contents of TGF-β1, VEGF, SDF-1, IGF-1, FMOD and IL-4 in tECM-DBTS were significantly higher than those in DBTS (Fig. 2d–i).

### tECM-DBTS promotes BMSCs proliferation and migration

**Fig. 3:**
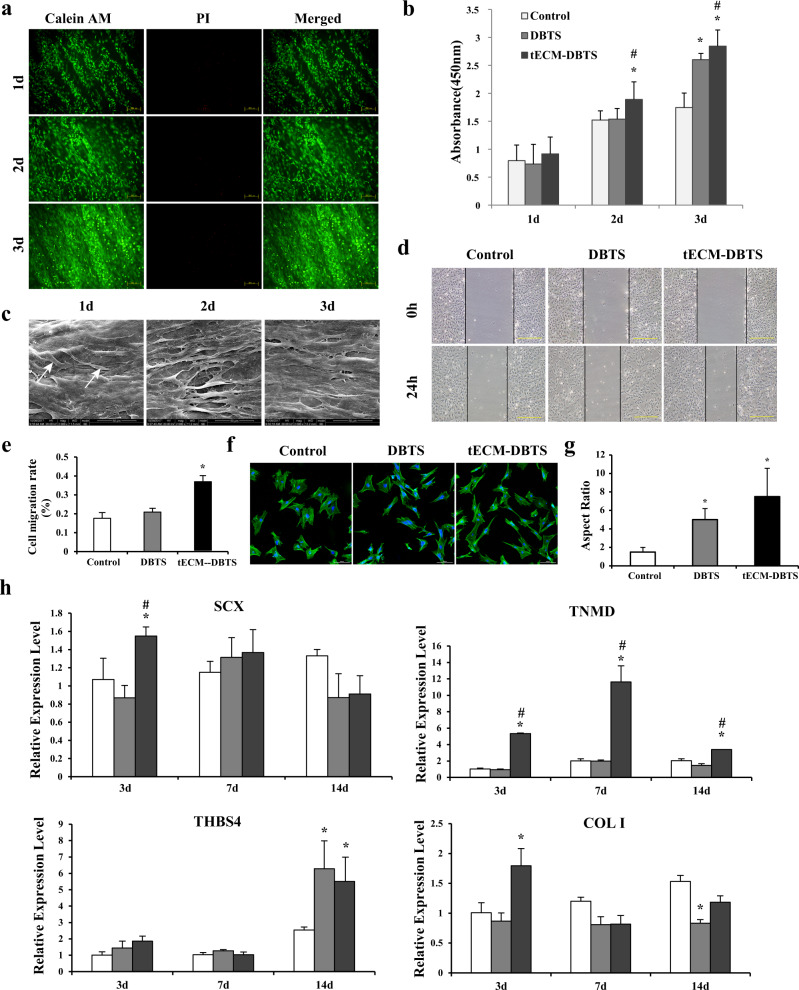
Characterization of the tECM-DBTS scaffold. **a** LIVE/DEAD staining analysis of BMSCs seeded on the tECM-DBTS at 1 day, 2 days and 3 days, with live cells stained green (calcein AM) and dead cells stained red (PI). **b** CCK-8 assay evaluating the effects of the control, DBTS and tECM-DBTS on BMSCs proliferation at 1 day, 2 days and 3 days (*n* = 4). **c** Surface ultrastructure of BMSCs seeded on the tECM-DBTS at 1 day, 2 days and 3 days. The white arrow showed that the BMSCs on the tECM-DBTS. **d** Scratch migration assay showing the effects of the control, DBTS and tECM-DBTS on BMSCs migration at 24 h. **e** Statistical results of cell migration rate of each group (*n* = 4). **f** Phalloidin staining displaying the effects of the control, DBTS and tECM-DBTS on BMSCs morphology at 2 days. **g** Statistical results of aspect ratio on BMSCs (*n* = 4). **h** Quantitative real-time PCR analysis of tenogenic-related gene expression of the control, DBTS and tECM-DBTS on BMSCs tenogenic differentiation at 3 days, 7 days and 14 days (*n* = 4). *Signifies a *p* < 0.05 as compared to the control. #Signifies a *p* < 0.05 as compared to the DBTS. Scale bars, (**a**): 200 μm; (**c**): 50 μm; (**d**): 500 μm; (**f**): 100 μm.

Live/dead cell staining revealed that the BMSCs exhibited good adhesion ability and cell viability at 1 day. With the extension of time, the cells maintained high cell viability on the surface of the tECM-DBTS, and only a small number of dead cells were visible (Fig. 3a). Statistical analysis of CCK-8 showed that the tECM-DBTS group exhibited a higher cell proliferation rate than the other two groups (Fig. 3b). Meanwhile, the growth of BMSCs on the tECM-DBTS was observed by SEM at different time points. Only a few cells were seen at 1 day. Then, numerous BMSCs were closely packed together to form a dense cell layer that covered the scaffold almost completely at 3 days (Fig. 3c). Overall, these results validated that tECM-DBTS was able to support the survival and proliferation of BMSCs.

The scratch migration assay of BMSCs showed relatively faster cell migration in the tECM-DBTS group than in the DBTS and control groups at 24 h (Fig. 3d). The semi-quantitative results indicated that the cell migration rate in the tECM-DBTS group was significantly higher than that in the DBTS or control group at 24 h (Fig. 3e). In short, these results demonstrated that tECM-DBTS promoted the migration of BMSCs.

### tECM-DBTS affects BMSCs morphology

As the time was extended to 24 h, cells exhibited elongated spindle-shaped morphology on tECM-DBTS and an elliptical morphology on DBTS and the control (Fig. 3f). Cell aspect ratios were quantified using actin-stained cell images to further quantify cell elongation. The aspect ratio of BMSCs on tECM-DBTS reached nearly 8, which was significantly higher than that on control and DBTS (Fig. 3g).

### tECM-DBTS facilitates BMSCs tenogenic differentiation

The expression level of SCX was significantly upregulated in BMSCs cultured in the tECM-DBTS group at 3 days but not at 7 or 14 days compared with the control and DBTS groups. TNMD, a tendon-specific marker gene, showed that the expression levels of the tECM-DBTS group were significantly higher than those of the control and DBTS groups at 3 days, 7 days and 14 days, respectively. After culture for 14 days, BMSCs on the DBTS and tECM-DBTS groups produced higher mRNA expression levels of THBS4. The expression of COL I was elevated significantly at 3 days in BMSCs cultured on the tECM-DBTS group, although there was no significant difference among the three groups at 7 days (Fig. 3h). As a whole, these data indicated that the tECM-DBTS was capable of promoting tenogenic differentiation of BMSCs.

### tECM-DBTS recruits exogenous and endogenous stem cells in vivo

**Fig. 4:**
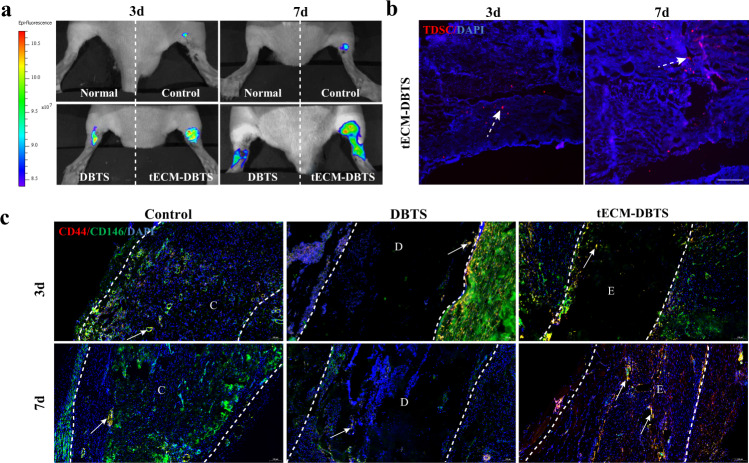
The ability of the tECM-DBTS to recruit exogenous or endogenous stem cells in vivo. **a** In-vivo tracking of tail vein injection PKH67-labeled exogenous TDSCs using IVIS analysis at 3 days and 7 days. Normal: normal tendon, Control: autologous tendon repair. **b** Fluorescent image of the TDSCs (red) existed at the Achilles tendon healing site of the tECM-DBTS at 3 days and 7 days. White dashed arrows indicate PKH67-labeled TDSCs. **c** Immunofluorescent staining of CD44 (red immunofluorescence), CD146 (green immunofluorescence), and DAPI (blue immunofluorescence) in the control, DBTS and tECM-DBTS groups at 3 days and 7 days. The white dashed lines indicate the interface between the scaffold and host tissues. White arrows indicate recruited stem cells. C: autogenous tendon repair; D: DBTS; E: tECM-DBTS. Scale bars, b: 200 μm; c: 100 μm.

TDSCs labeled with PKH67 before tail vein injection were used to track exogenous stem cells migration. At 3 and 7 days after injection, IVIS images showed that a slightly more positive PHK67 signal was detected at the Achilles tendon healing site in the tECM-DBTS group than in the DBTS group, both of which were significantly more positive signals than in the control group (Fig. 4a). Normal tendon was used as a normal control group (Normal). In addition, fluorescence section showed that a higher number of PKH67-labeled cells existed at the Achilles tendon healing site at 7 days than at 3 days for tECM-DBTS (Fig. 4b).

At the cellular level, tECM-DBTS induced recruitment of CD146/CD44 double positive endogenous stem cells to the repair region of the tendon. However, no apparent CD146/CD44 double positive endogenous stem cells were observed in the DBTS group. In the control group, there were many CD146 positive endogenous stem cells. However, the DBTS and tECM-DBTS groups showed significantly higher CD44 positive cell density than the control group (Fig. 4c). These results confirmed that tECM-DBTS has the ability to recruit endogenous stem cells.

### Early macrophage response to tECM-DBTS

**Fig. 5:**
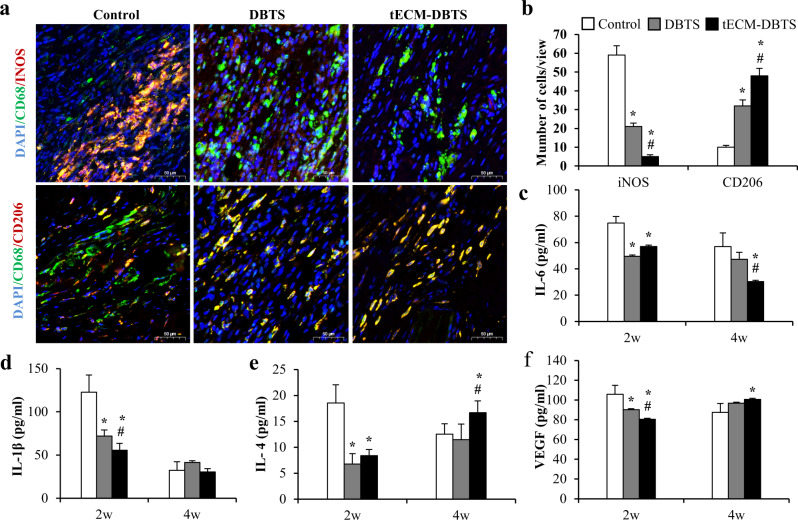
Macrophage response during early remodeling stage. **a** Double staining showed the distribution of the CD68 positive pan-macrophage (green) and CD206-positive M2 type macrophages (red), or iNOS-positive M1 type macrophages (red) at 2 weeks. **b** Amount of CD68, CD206 and iNOS-positive cells was computed (*n* = 5). The dynamic changes of cytokines and angiogenic factor expression were detected by ELISA, including IL-6 (**c**), IL-1β (**d**), IL-4 (**e**), VEGF (f) (*n* = 3). *Signifies a *p* < 0.05 as compared to the control. ^#^Signifies a *p* < 0.05 as compared to the DBTS. Scale bars, a: 50 μm.

As shown in Fig. 5a, immunofluorescence double staining for CD68/CD206 and CD68/iNOS showed the lowest number of iNOS-positive proinflammatory M1 macrophages and the largest number of CD206-positive anti-inflammatory M2 macrophages in the wounds at the early point of 2 weeks in the tECM-DBTS group. There were predominantly iNOS-positive macrophages in the control group. In the DBTS group, M1 macrophages were distributed around the graft, which was confirmed by high levels of CD68 and iNOS expression at 2 weeks (Fig. 5b). Quantification of the M1 (iNOS)/M2 (CD206) macrophage ratio in the wounds showed that tECM-DBTS was significantly lower than DBTS and the control group (Supplementary Fig. 1).

To further evaluate the dynamics of inflammation-associated cytokine expression in the early stages of tendon repair, we measured the expression of inflammation-related cytokines at 2 and 4 weeks. Pro-inflammatory cytokines, including IL-6 and IL-1β, and anti-inflammatory cytokines, including IL-4, were analysed. The expression of IL-6 was reduced at 4 weeks compared to 2 weeks post-surgery in the tECM-DBTS group (Fig. 5c). The expression of IL-1β in the tECM-DBTS group was significantly decreased compared with that in the control and DBTS groups at 2 weeks (Fig. 5d). The expression of anti-inflammatory IL-4 in tECM-DBTS was significantly higher than that in DBTS and control groups at 4 weeks (Fig. 5e). During the inflammatory phase, M2 macrophages can also release VEGF and increase endothelial sprouting. The expression of VEGF in the tECM-DBTS was significantly higher than that in the DBTS and control groups at 4 weeks (Fig. 5f).

### Evaluation of regenerated tendons in the healing site

Immunofluorescence staining of TNMD was conducted (Supplementary Fig. 2), and the tECM-DBTS group displayed significantly higher expression of TNMD at 2 weeks. This result indicated that tECM-DBTS significantly enhanced the expression of tendon related proteins at 2 weeks.

**Fig. 6:**
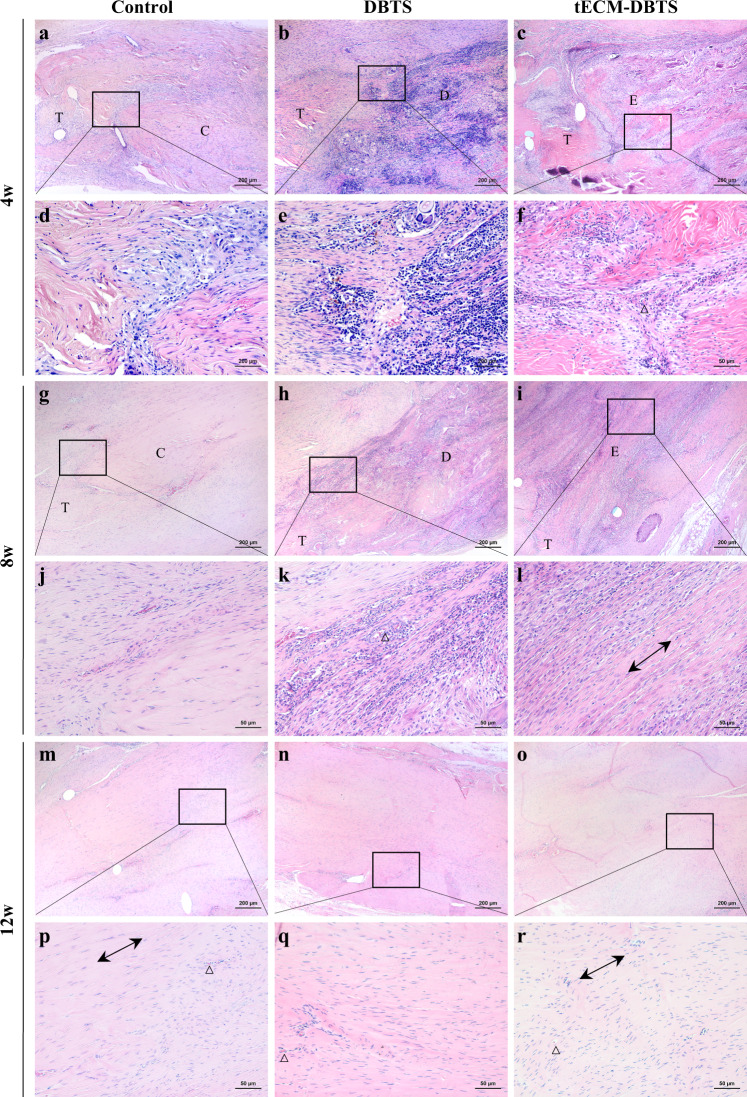
Representative HE staining of tendon regeneration in a rat Achilles tendons defect model of the control, DBTS and tECM-DBTS group. **a**–**f** Postoperative 4 weeks, (**g**–**l**) 8 weeks and (**m**–**r**) 12 weeks. The black box is a magnification of the image. Δ represents capillaries. The bidirectional arrows indicate the direction of collagen fiber alignment. T: Achilles tendon of rat; C: autogenous tendon repair; D: DBTS; E: tECM-DBTS. Scale bar, (**a**, **b**, **c**, **g**, **h**, **i**, **m**, **n**, **o**) 200 μm; (**d**, **e**, **f**, **j**, **k**, **l**, **p**, **q**, **r**): 50 μm.

The results of H&E staining were shown in Fig. 6. At 4 weeks post operation, all three groups featured fiber rearrangement and infiltration of inflammatory cells. In comparison with the control group, a higher degree of inflammation was visible in DBTS and tECM-DBTS (Fig. 6a–f). Vascularization was visible at the repair region of the tendon. Immunofluorescence staining of CD31 and VEGF suggested that capillaries were distributed within the tECM-DBTS, and tECM-DBTS increased the angiogenic ability compared with DBTS at 4 weeks (Supplementary Fig. 3). At 8 weeks post operation, the inflammatory response was decreased in the tECM-DBTS group compared with that at 4 weeks. The tECM-DBTS group had better aligned collagen fibers than the DBTS group (Fig. 6g–l). At 12 weeks post operation, only little inflammation could be detected in healing tendons. The tECM-DBTS was replaced by neo-tendon, and defect regions were barely visible. In addition, the morphology, ECM deposition and cellularity of neo-tendons in tECM-DBTS were similar to those of the control group (Fig. 6m–r). Gross observation at 12 weeks also confirmed that there was no significant difference in appearance between the control group and the tECM-DBTS group (Supplementary Fig. 4).

**Fig. 7:**
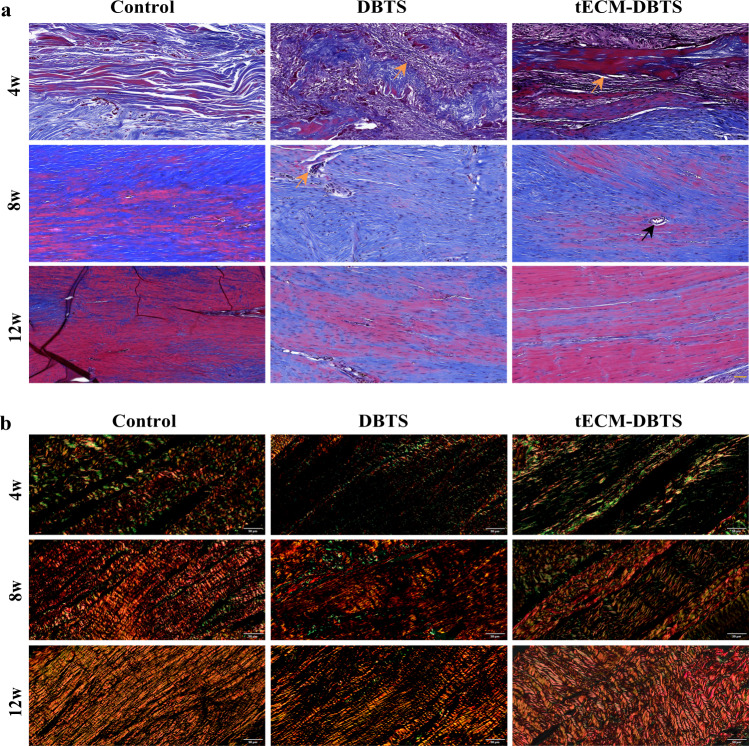
tECM-DBTS enhances collagen remodeling in a rat Achilles tendons defect model. **a** Representative Masson’s trichrome staining of tendon regeneration in the control, DBTS and tECM-DBTS groups at 4 weeks, 8 weeks and 12 weeks. Orange arrows indicate the scaffolds, black arrows indicate the vascularization. **b** Representative polarized light images showing the collagen type at the repaired tissue site. Green indicates type III collagen and red indicates type I collagen. Scale bars, (**a**): 50 μm; (**b**): 50 μm.

Masson staining further revealed the tissue morphological changes of collagenous tissue reorganization. Scaffold that was not completely degraded was observed in the DBTS group at 8 weeks. The newly formed tissues at 12 weeks after surgery were seen to have two types of collagen fibers, mature (stained red) and immature ones (stained blue). It is apparent that the mature collagen fibers in the tECM-DBTS group were more enriched than those in the DBTS group (Fig. 7a). Sirius red staining showed that type III collagen (stained green) was gradually replaced by type 1 collagen (stained red) over time. Abundant extracellular matrix type I collagen was deposited with orderly alignment in the tECM-DBTS group, while the DBTS group showed less type I collagen deposition at 12 weeks after surgery (Fig. 7b). These results demonstrate that tECM-DBTS provided a good microenvironment for tendon regeneration similar to autogenous tendons.

### Functional and biomechanical evaluation of the repaired tendon

**Fig. 8:**
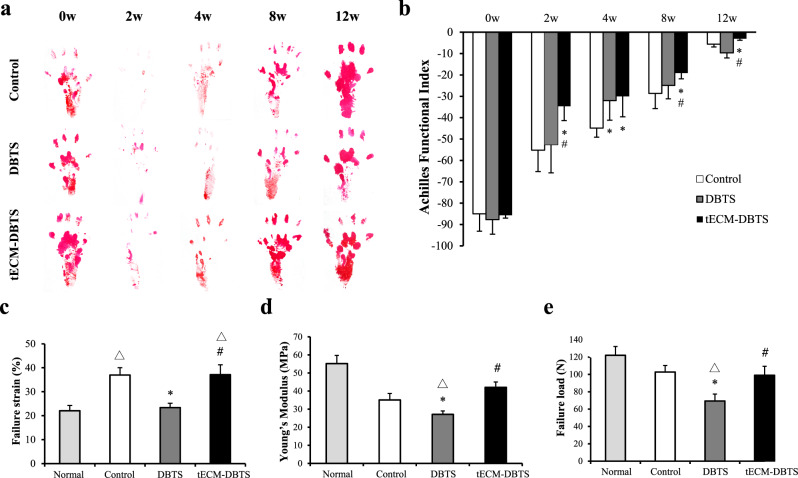
Functional and biomechanical testing of the control, DBTS and tECM-DBTS implant. **a** The paw prints of the control, DBTS and tECM-DBTS group pre-operative (0w) and post-operative at 2, 4, 8 and 12 weeks. **b** Functional testing of the Achilles tendon over 12 weeks was performed to assess recovery of motion (*n* = 4). **c** The failure strain, (**d**) Young’s modulus and (**e**) failure load of the different groups (*n* = 4). Δ Signifies a *p* < 0.05 as compared to the normal tendon. *Signifies a *p* < 0.05 as compared to the control. ^#^Signifies a *p* < 0.05 as compared to the DBTS.

The hind paw prints were narrower post-surgery in all groups at 2 weeks. The hind paw prints of rats in the DBTS group were broadly narrower and longer than those of rats in the tECM-DBTS group from 2 weeks onwards (Fig. 8a). The tECM-DBTS group showed significant recovery of tendon AFI at 2 weeks and showed continued improvement until it returned to AFI values at 12 weeks, which were comparable to pre-injury status values (week 0). Tendon AFI scores remained consistently higher in the tECM-DBTS group than in the DBTS group, with significant differences observed at 8 and 12 weeks (Fig. 8b). In biomechanical testing, all specimens ruptured at the surgical repair site. Failure strain in the tECM-DBTS group had a significantly higher value compared with the DBTS group, while no significant difference was found between the tECM-DBTS and control groups (Fig. 8c). Meanwhile, the Young’s modulus and failure load of tECM-DBTS group were significantly higher than those of the DBTS group, and no significant difference was found between tECM-DBTS and normal groups at 12 weeks (Fig. 8d, e).

## Discussion

Repair of tendon injuries poses significant clinical challenges due to insufficient spontaneous regeneration and high biomechanical demands, underscoring the importance of developing tissue engineering strategies for functional tendon repair. Recently, biomechanical and biochemical-functional augmentation has been recommended for tendon repair, particularly for large or massive tears^43^. The tendon-healing process incorporates biochemical and biomechanical responses to sustain physiological loading^44^. However, currently available augmentation materials have not yielded satisfactory results^45^. Fortunately, our group fabricated a DBTS scaffold that exhibited matching mechanical strength and excellent suture retention strength, which provided a mechanically-functional microenvironment for tendon repair^38^. Further studies confirmed that the extracellular matrix components secreted by TDSCs contain bioactive factors that regulate the migration of stem cells^31^ and comprise proteoglycans that determine tenogenic differentiation^30^. On this basis, we combined the DBTS with extracellular matrix from TDSCs to fabricate a biomechanically and biochemically functional scaffold (tECM-DBTS), to provide a functional and stem cells’ ECM-based microenvironment for tendon regeneration. It was found that the tECM-DBTS increased the content of bioactive factors and had a better performance for the proliferation, migration and tenogenic differentiation of BMSCs compared to DBTS. Furthermore, our results demonstrated that the tECM-DBTS provides a microenvironment to facilitate the recruitment of endogenous stem cells to the region of injury, rather than traditional tissue engineering that requires reliance on seeding cells and in vitro engineered constructs^32^.

It has been reported in a previous study that the unique biomechanical properties of tendons are largely attributed to the high degree of organization of the structure^46^. Consequently, when the tendinous membrane (endotenon and epitenon) or fascicle of DBTS is complete, it may maintain the inherent mechanical property to support functional movement in large-to-massive tendon repair^38^. Our in vitro analyses confirmed that the tECM-DBTS scaffold still retained the ultrastructure of the native tendon very well with a mild decellularization method. Therefore, the tECM-DBTS scaffold displays an appropriate suture and fixation with the host tendon to transfer mechanical stimuli, which plays a key role in tenogenic differentiation^16^. It has been reported that the biomechanical matching to tendon tissue specificity and a dynamic mechanical stimulus induce morphologic changes in stem cell and increase the expression of tendon-related genes^16^.

Although the mechanical properties of the ECM have been shown to have an important influence in guiding stem cell differentiation, it is not the only contributing factor^12^. Our study showed that the contents of bioactive factors (TGF-β1, SDF-1, FMOD and IL-4) were significant decreased in DBTS when compared with those in NTS (Supplementary Fig. 5). Here, tECM-DBTS was expected to enhance bioactivity by increasing bioactive factors while retaining the mechanical properties of tendons to design microenvironments that mimic the stem cell niche driving cells towards their preferred lineages. In order to retain as many bioactive factors as possible, a mild decellularization method was used in this study to perform the decellularization treatment. Meanwhile, such a decellularization method has been reported in other study^47^. Promisingly, after removal of the cells in these tendons, the resultant scaffold still retained excellent preserved ultrastructure and biochemical components of native tendon ECM. Our histological results and SEM images showed that the ECM produced by TDSCs was effectively bound to the DBTS after decellularization. Furthermore, the bioactive factors of the ECM secreted by TDSCs, including VEGF, TGF-β, SDF-1, IGF-1 and FMOD, were of particular interest. It is known that these bioactive factors perform multiple functions during tendon regeneration. As expected, the content of these bioactive factors was significantly improved in tECM-DBTS when compared with the bare DBTS. TGF-β is one of the cytokines that plays a role in all phases of wound healing^48,49^. It has been proved to facilitate the proliferation and the synthesis of ECM of the fibroblasts^50^. The primary roles of IGF-1 seem to be to stimulate the proliferation of fibroblasts and other cells at the region of injury, and to subsequently increase the production of collagens and other extracellular matrix components in these cells during the remodeling phase^51^. SDF-1 is an important chemokine that promotes the migration of BMSCs^52^. These results implied that the bioactive factors retained in the tECM-DBTS scaffold may be biologically active, thus favoring the proliferation and migration of the stem cells. Previous studies have shown that FMOD, as an important component of the extracellular matrix of tendons, determined the tenogenic differentiation of TDSCs^30^. Additionally, the results of our RT-qPCR analysis indicated that the tenogenic differentiation-related genes (SCX and TNMD) were significantly upregulated in the tECM-DBTS group. Regarding BMSCs, an aspect ratio value of >8 could significantly inhibit osteogenic differentiation^45,53^. Our results suggest that BMSCs on tECM-DBTS showed an elongated cell shape with an aspect ratio value of 7.49 ± 3.05. Collectively, our findings indicate that the tECM-DBTS supplies suitable microenvironments to enhance the tenogenic differentiation of seeded BMSCs. What needs to be pointed out is that six representative bioactive factors were detected in this study. There should be some other bioactive factors in the tECM-DBTS, which also may influence the biological functions of BMSCs.

As a traditional tissue engineering strategy, many cells seeded on the scaffold are potentially ineffective when implanted into an injured environment characterized by hypoxia, intense inflammation and nutritional deficiency^54^. Therefore, researchers hope to use the inductive ability of the scaffold itself to recruit and functionalize the innate regenerative capacity of endogenous stem/progenitor cells and enable in situ tendon repair^55,56^. In the current study, we used tECM-DBTS as a biochemically functional scaffold to overcome the disadvantages of conventional tissue engineering techniques and provide a strong experimental basis for the research and development of tissue-engineered tendon biologic scaffolds with tissue-inducing properties. Interestingly, we were able to visualize the CD146/CD44 double positive stem cells, within the tECM-DBTS graft at 7 days post-implantation. These results suggest that the tECM-DBTS could recruit the endogenous stem cell to the region of injury after surgery. The cause of this phenomenon may be due to the rise of SDF-1 in the tECM-DBTS scaffold. SDF-1 is essential for cell migration and recruitment of host stem cells to injured tissues^57^. The tECM-DBTS enhances biochemical function of recruiting endogenous stem cells by increasing the amount of SDF-1. Previous studies have demonstrated that CD146^+^ TDSCs exist in the original and neo-tissue of tendon^58^. One can speculate that these early infiltrating cells may be TDSCs. The results are also consistent with previous reports that the migration of TDSCs to the injury region is important for tendon-healing process^59^. We were able to visualize the high expression of TNMD within the tECM-DBTS scaffold at 2 weeks post-implantation. TDSCs mature into tenocytes and are present in the neo-tendon, which could explain the high expression of TNMD at 2 weeks. Notably, cell tracking analysis indicated that tail vein injected PKH67-labelled TDSCs were recruited near the site of tendon injury. Fortunately, the tECM-DBTS group had stronger fluorescence than the control and DBTS group, indicating that the tECM-DBTS group had a better ability to recruit PKH67-labelled TDSCs. More importantly, the repaired tendon was harvested and then frozen sectioned to further determine the number of labelled cells present in the Achilles tendon. We found that there were PKH67-labelled TDSCs in the repaired tendon. Additionally, in-vivo histological staining results showed that the Achilles tendon in the tECM-DBTS group regenerated significantly better than that of DBTS group. Based on our results, we speculate that tECM-DBTS scaffold could accelerate the regeneration of Achilles tendon by recruiting endogenous stem cells and participating in the functionalization of these stem cells.

Numerous studies have shown that biomaterial-based scaffold for immunomodulation can promote a positive remodeling response by inducing macrophage polarization^60,61^. In our study, Achilles tendon defect repair in the tECM-DBTS group accumulated macrophages with higher marker expression of CD206, which was M2 macrophage (anti-inflammatory). When macrophages are induced to polarize into M2 phenotype, they secrete cytokines and chemokines that promote regeneration. In the present study, our results implied that tECM-DBTS possessed the stronger and faster M2 polarization of macrophages than DBTS. Actually, both DBTS and tECM-DBTS, composed mainly of decellularized tendon, are extracellular matrix scaffolds which have been shown to promote a switch from a predominantly M1 phenotype immediately following implantation to a population enriched in M2 phenotype^62^. When these scaffolds are degraded, they release growth factors, cytokines, and cryptic peptides that can promote M2 polarization of macrophages^63^. Most notably, our tECM-DBTS combined the DBTS with extracellular matrix from TDSCs. This stem-cell derived ECM is rich in a variety of bioactive innate factors, many of which are known to affect macrophage polarization^64^, including anti-inflammatory cytokines^41^. Recent works have reported that macrophages would express arginase, CD206, CD163, IL-10, and IL-1 receptor antagonist when they were stimulated with IL-4, and would assume an M2 phenotype^65,66^. Based on this, we speculated the significantly enhanced IL-4 is the most likely origin of M2 polarization caused by tECM-DBTS, but we didn’t examine the possible mechanisms. Consistent with these findings, a recent study showed that ECM from TDSCs can drive macrophage polarization toward the anti-inflammatory M2 phenotype and that M2 macrophages educated by implanted ECM scaffolds can promote early tendon healing^32,67^.

**Fig. 9:**
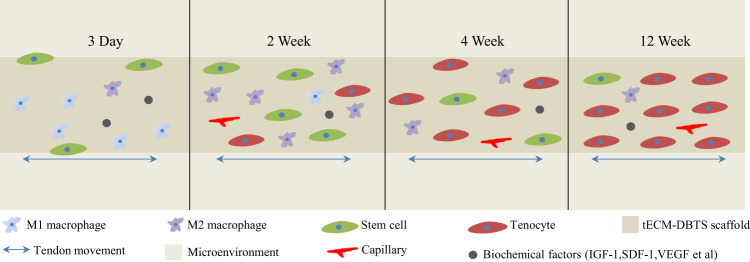
Schematic illustration of the tendon regeneration process of the tECM-DBTS scaffold in vivo. Changes in tendon at different time points were described.

Our results highlight the potency of the biomechanical and biochemical functions for regenerating tendon tissue. Based on the tendon regeneration process of tECM-DBTS in vivo (Fig. 9), we found that the tECM-DBTS group could improve tissue regeneration and promote tendon healing more than the DBTS group. A large number of inflammatory cells were infiltrated into the tECM-DBTS at 4 weeks after surgery during the inflammation phase, which is consistent with a previous investigation showing that a modest inflammation reaction is necessary to trigger an appropriate healing response^68^. Between 4 and 8 weeks, the inflammatory response was relieved and the scaffold was remodeled into an organized and collagen-rich ECM. VEGF is an important angiogenic factor to influence the vasculature and angiogenesis^69^. In this study, the tECM-DBTS group exhibited increased angiogenic ability in vivo, as determined by immunofluorescence assay, which should be mainly due to the increased secretion of angiogenic growth factors such as VEGF in tECM-DBTS, as confirmed by ELISA analysis. Petersen et al. demonstrated that VEGF is involved in the process of ACL reconstruction and angiogenesis, which would contribute to ACL remodeling^70^. At 12 weeks, ECM composition, fiber rearrangement at the junction and cellularity similar to those of the control group. The tECM-DBTS displayed a more mature and well-aligned collagen matrix at 12 weeks, which can lead to further mechanical augmentation and decrease the risk of re-rupture. No fractures or ruptures were observed in our tECM-DBTS scaffold, indicating that the scaffold could withstand dynamic loading during this early remodeling process. The data presented in this study suggested that the rate of neo-tissue regeneration was fast enough to compensate for the loss of mechanical properties caused by material degradation. This deduction was further reinforced by the functional assessment of the AFI. The application of IGF-I has been proven to increase in the AFI of injured rat tendon^71^. Accordingly, the mechanical properties of tendons are mainly contributed by the well-aligned collagen fibers, and the tECM-DBTS group showed favorable mechanical properties. Further augmentation of ECM bioactivity via mechanical stimulation, should provide a rational basis for better utilizing ECM bioactivity for tendon repair. Correspondingly, the excellent mechanical properties of the tECM-DBTS group were ultimately marked for the high AFI. Most importantly, compared to cell-seeding methods, our tECM-DBTS scaffold can not only be stored long-term without a mechanical strength decrease but also has elemental mechanical strength, inherent ultrastructure and elevated bioactive factors. These cues act synergistically to design microenvironments that mimic the stem cell niche driving cells towards to tendon differentiation. In this study, we developed a decellularized tendon scaffold modified by ECM secreted from tendon-derived stem cells for tendon regeneration. The tECM-DBTS scaffold retained the architecture of the native tendon and showed biomechanical matching to sustain tendon movement. Furthermore, the tECM-DBTS scaffold increased the content of bioactive factors and was better capable of supporting the proliferation of BMSCs as well as promoting the migration and tenogenic differentiation of BMSCs in vitro. Our results demonstrate that the tECM-DBTS scaffold promotes tendon regeneration and improves the mechanical properties of Achilles tendon defects in rats by recruiting endogenous stem cells and participating in the functionalization of these stem cells. Overall, we believe that this scaffold constructs a microenvironment including biomechanical and biochemical cues, which is more conducive to stem cell differentiation and tendon regeneration for large-to-massive defects.

This study has the following limitations. First, we only examined the genes of tenogenic differentiation in vitro. Therefore, our results may not reveal the full spectrum of the function of the tECM-DBTS scaffold in tendon healing. Expression of tenogenic differentiation proteins may be needed in future studies. Second, although many different types of bioactive factors may be preserved in tECM-DBTS, only six representative factors IGF-1, SDF-1, VEGF, TGF-β1, FMOD and IL-4, were studied. In future studies, we should focus on more kinds of bioactive factors and the effects on the functionalization of endogenous stem cells. Third, our results could not fully reflect the tenogenic differentiation of the recruited stem cells, and a more appropriate characterization method should be selected in the future. Furthermore, we only inferred the possible cause of tECM-DBTS-induced polarization of macrophages toward M2 type, and we will further elucidate the underlying mechanism of M2 polorization caused by tECM-DBTS in our future research.

## Methods

### Ethics statement

The experimental protocol for the use of SD rats in this study was conducted in accordance with the approved guidelines set out by the Animal Ethics Committee of West China Hospital of Sichuan University (NO. 2019293 A). Male SD rats were obtained from Dashuo Experimental Animal Center (China). Animals were housed in ventilated cages on a 12:12-hour light/dark cycle with ad libitum access to food and water.

### Isolation and culture of rat TDSCs and BMSCs

Two-week-old Sprague Dawley (SD) rats’ TDSCs and BMSCs were used in this study. Both TDSCs and BMSCs were isolated and cultured as previously described^29^. Briefly, TDSCs were isolated from the rat Achilles tendons and flexor tendons by digesting with 3 mg/ml collagenase type I (Worthington) and 4 mg/ml dispase (Sigma) for 50 min at 37 °C. The tissues-enzyme solution was filtered with a 70 μm cell strainer (BD, Falcon, USA) to obtain a single cell suspension. The released cells were resuspended in complete medium supplemented with 15% FBS. To isolate the BMSCs, bone marrow was flushed out from femoral marrow cavities with LG-DMEM. Then, the suspension was filtered through a 70 μm cell strainer and centrifuged at 1200 rpm for 5 min. Both BMSCs and TDSCs were grown in Dulbecco’s modified Eagle’s medium (DMEM, Gibco) supplemented with 15% fetal bovine serum (FBS), 100 U/ml penicillin, 100 mg/ml streptomycin, and 2 mM L-glutamine (all from Invitrogen, Carlsbad, CA) at 37 °C with 5% CO_2_. Cells of passage 3 were used in this study.

### Fabrication of the tECM-DBTS scaffold

The preparation process of DBTS was similar to our previous study^37^. The TDSCs were seeded at a density of 1 × 10^6^ cells/cm^2^ onto DBTS, and incubated with DMEM containing 10% FBS for 7 days at 37 °C in a 5% CO_2_ incubator. After 7 days of co-culture, the medium was replaced with DMEM containing 10% FBS and 50 μM ascorbic acid (vitamin C) at 37 °C in a 5% CO_2_ incubator for 8 days to obtain TDSCs-DBTS composites. Next, the composites were placed in decellularization solution (0.5% Triton X-100 with 20 mM ammonia) for 10 min, and then the solution was removed and washed 3 times with PBS (pH 7.4) for 30 min each time. Finally, the tECM-DBTS scaffold was obtained. Each sample of the tECM-DBTS was pruned to 8 mm long and 8 mm wide.

### Characterizations of the tECM-DBTS scaffold

The decellularization of the TDSCs-DBTS composites was evaluated with histology and DNA assays. For histology, the DBTS, TDSCs-DBTS and tECM-DBTS (*n* = 4 for each group) were fixed in 4% paraformaldehyde at room temperature for 24 h, embedded in paraffin, and sliced to a thickness of 5 μm using a microtome. The sagittal sections were deparaffinized, rehydrated and washed in distilled water, and then stained with hematoxylin and eosin (H&E, Sigma) and Masson’s Trichrome staining (Sigma). For DNA assays, the samples (*n* = 4 for each group) were digested with 0.1 mg/ml Proteinase K (Sigma) at 50 °C for 24 h. After digestion, samples were centrifuged and then the purified supernatant was extracted with phenol/chloroform/isoamyl alcohol (25:24:1, v:v). After ethanol precipitation and drying, the sample was rehydrated in 1 mL TE buffer. DNA contents were quantified by Picogreen DNA assay (Invitrogen) using 480 nm as the excitation wavelength and 520 nm as the emission wavelength on a Synergy H1 microplate reader.

The ultrastructure of the DBTS, TDSCs-DBTS and tECM-DBTS was visualized with scanning electron microscopy (SEM). The freeze-dried samples (*n* = 3 for each group) were fixed in 2.5% glutaraldehyde for 2 h at 4 °C. After dehydration with a graded ethanol series, the specimens were subjected to critical point drying after a sputter coating with gold. SEM images of cross sections were taken using an FEI Inspect F50-SEM (Netherlands) with a 20 kV acceleration voltage.

The bioactive factors retained in DBTS and tECM-DBTS were evaluated by enzyme-linked immunosorbent assay (ELISA). Soluble molecules were isolated from DBTS and tECM-DBTS (*n* = 4 for each group) using the Radio Immunoprecipitation Assay (RIPA) Lysis Buffer (Beyotime, China) containing protease inhibitor. The extraction lysate was centrifuged at 15,000 g for 30 min at 4 °C and then the supernatant was collected. ELISA measures were performed to examine TGF-β1, VEGF, SDF-1, FMOD, IGF-1 and IL-4 according to the manufacturer’s instructions (TGF-β1, DL-develop; VEGF, DL-develop; SDF-1, DL-develop; FMOD, Ruixin; IGF-1, DL-develop; IL-4, Zuocai). The contents of bioactive factors of native tendon sheet (NTS) and DBTS were evaluated by the same method.

### Responses of stem cells to the tECM-DBTS scaffold

Cell viability and proliferation were determined by the Live/Dead cell staining and CCK-8 assay, respectively. Briefly, BMSCs were cultured on the surface of tECM-DBTS at a density of 4 × 10^3^ cells/cm^2^ for 1, 2 and 3 days. The fluorescence of green (live cells) and red (dead cells) was observed using a fluorescence microscope (Zeiss, Germany). Then, the BMSCs harvested after 1, 2 or 3 days of incubation were incubated in 10% (v/v) CCK-8 solution for 2 h at 37 °C in a 5% CO_2_ incubator. Subsequently, 100 μL culture solution of each well was transferred to a new 96-well plate and the absorbance was detected at 450 nm using a Synergy H1 microplate reader.

The morphology of BMSCs on the tECM-DBTS scaffold was assayed by SEM and filamentous actin (F-actin) staining. To further quantify cell elongation, the aspect ratio of cells was measured with Image J software.

The effect of tECM-DBTS on the BMSCs migration in vitro was measured by the scratch migration assay. Aiming to avoid the effect of different scaffold surface microstructures on cell behavior, we prepared extracts from sterilized DBTS and tECM-DBTS by modifying the protocol according to previous publication.^72^ Briefly, the DBTS and tECM-DBTS were chopped and incubated in serum-free DMEM (10 mg/mL) for 72 h at 37 °C with 5% CO_2_. The supernatants were collected as extracts for later use. The BMSCs (1 × 10^5^ cells/ well) were seeded into 12-well plates and incubated for 12 h under serum starvation. A scratch was made with a sterile 200 μL pipet tip. After removing the medium, the wells were gently washed with PBS to eliminate the shed cells. The cells were then exposed to the extract of L-DMEM (control group), DBTS and tECM-DBTS, respectively. The images were taken at 24 h after migration. The cell migration rate (%) was calculated as follows: (the width of the original scratch − the width of the actual scratch) / the width of the original scratch × 100%.

The genes related to tenogenic differentiation (*n* = 4 for each group) were measured after co-culture of BMSCs with tECM-DBTS for 3 days, 7 days, and 14 days. Total RNA was extracted by lysing the cells using TRIzol reagent (Invitrogen, Carlsbad, CA, USA) following the manufacturer’s protocol. The mRNA was reverse transcribed using GoScript Reverse Transcription System (Takara, Japan). The synthesized cDNA was amplified by quantitative TaqMan RT-qPCR. Rat tendon-related genes, including scleraxis (SCX), tenomodulin (TNMD), thrombospondin-4 (THBS4), and collagen types I (COL I), as well as the internal control, Glyceraldehyde-3-phosphate dehydro-genase (GAPDH) were synthesized by Qingke Biotech. The sequences of the primers were listed in Table 1. The expression of target gene was normalized to that of GAPDH gene.

**Table 1:** Primer sequences, product size and annealing temperature used for PCR analysis.

Genes	5’-3’ Primer and probe sequences	Production size (bp)	Annealing temperature (°C)
GAPDH	Forward GCAAGTTCAACGGCACAG Reverse GCCAGTAGACTCCACGACAT	140	60
SCX	Forward AGAACACCCAGCCCAAACA Reverse GTGGACCCTCCTCCTTCTAAC	111	59
TNMD	Forward GGACTTTGAGGAGGATGG Reverse CGCTTGCTTGTCTGGTGC	128	57
THBS4	Forward AATACCATCCCTGCTACCC Reverse TTCCGACACTCGTCAACA	163	60
COL I	Forward CGAGTATGGAAGCGAAGG Reverse AGTGATAGGTGATGTTCTGG	101	59

### Animal experiment

Sixty male Sprague-Dawley rats (300–320 g) were operated bilaterally according to the previous scheme with some modifications^45^. After anesthesia by pentobarbital sodium (40 mg/kg), a longitudinal lateral skin incision was made, and a defect of 6 mm was created on Achilles tendons. Rats used in the animal experiment were randomly allocated into three groups as follows: Control group (autogenous tendon repair), DBTS group, and tECM-DBTS group. The suitably sized grafts for different groups were sutured in an end-to-end way using 6–0 monofilament nylon sutures (Ethicon, USA), respectively. The wounds were closed with 3–0 monofilament nylon sutures (Ethicon, USA). Penicillin (10,000 U/kg) was injected for infection prevention after surgery. Each rat was returned to its cage for normal activity without fixation. At predetermined time points after operation, rats without injection of exogenous TDSCs were sacrificed and the grafts together with surrounding tissues and part of the host’s Achilles tendon tissue were harvested for histological and immunofluorescence analysis. At week 12, rats from each group were euthanatized and the muscle-Achilles tendon connected to the calcaneus was harvested for biomechanical testing.

### TDSCs labeling and tracking

To individually trace the migration of exogenous TDSCs in rats (*n* = 3 rats per group), the PHK67 labeled TDSCs were used through a tail vein. Operation method was the same as described above. After operation, the PKH67-labeled TDSCs (1 × 10^7^ cells) in 100 μl PBS were immediately injected into rats via tail vein. Meanwhile, the normal Achilles tendon (Normal group) was set to exclude the influence of fur glow. A non-invasive tracking system (IVIS Spectrum, PerkinElmer, USA) was used to image the PKH67 intensity and distribution on the rat Achilles tendon at the time point of injecting the cells 3 days and 7 days later. Furthermore, the repaired tendon was harvested and then frozen sectioned to further determine the number of labeled cells present in the Achilles tendon tissue, while DAPI was used to stain the cell nuclei.

### Histological and immunofluorescence analysis

At 2, 4, 8 and 12 weeks after surgery, samples (*n* = 5 for each group) were fixed in 4% paraformaldehyde at 4 °C for 24 h and dehydrated through an alcohol gradient before being embedded in paraffin wax. Longitudinal sections (5 μm in thickness) were prepared and then stained with H&E, Masson’s trichrome staining and Sirius red staining according to the manufacturer’s protocol. Micrographs from sections were captured at the suture regions. Three randomly selected visual fields of each section were captured by an optical microscope (Zeiss, Germany). For immunostaining, longitudinal sections were stained with primary antibodies purchased from Abcam (Cambridge, UK): anti-CD68 antibody (1:100; ab31630) to observe inflammatory cells; and anti-mannose receptor anti-CD206 (1:20000; ab64693) and anti-iNOS antibody (1:400; ab15323) to visualize M2 and M1 macrophages, respectively. Surface markers were stained in the same way using anti-CD146 (1:200; ab75769) and anti-CD44 (1:200; ab157107) to observe the recruitment of endogenous stem cells. Anti-TNMD antibody (1:200; bs-7525R) was stained to characterize the tenogenic differentiation of stem cells during the repair process. Anti-CD31 antibody (1:100; ab28364) and anti-VEGF antibody (1:200; YM3681) were used to visualize the vascularization of the implanted scaffold. The secondary antibodies, purchased from Invitrogen were incubated with sections for 1 h at 37 °C. Nuclei were counterstained with DAPI solution. Images were taken using a fluorescence microscope (Zeiss, Germany), and the number of positive cells in the sections (a random field of vision in each section) was analyzed by ImageJ software.

### Inflammation-related cytokine examination

To further assess the dynamic changes in inflammation-related cytokines during the early stages of tendon repair, we measured the expression of anti-inflammatory (interleukin-4, IL-4), pro-inflammatory (interleukin-6, IL-6 and interleukin-1β, IL-1β) and VEGF at 2 and 4 weeks. The repaired tendons with surrounding tissues were harvested (*n* = 3 for each group) to measure the inflammation-related cytokines using the ELISA for Premixed Panels and a diluent kit. The ELISA assay procedures were performed as described above.

### Measurement of Achilles functional index (AFI)

At 0, 2, 4, 8 and 12 weeks after surgery, the Achilles tendon functional test was performed according to Murrell’s method^73^. A walkway covered with white paper, 20 cm × 80 cm, was used for functional testing. The right hind paws of the rats (*n* = 4 for each group) were painted with a red inkpad and then rats could walk along the walkway. The paw prints were scanned and relevant parameters, such as print length, print width (distance between first and fifth toe) and intermediate toe width (distance between second and fourth toe) were measured. The print length factor (PLF), toe spread factor (TSF) and intermediate toe factor (ITF) were then calculated using ImageJ software v1.5 (NIH). AFI was determined according to the formula: AFI = 74(PLF) + 161(TSF) + 48(ITF) − 5.

### Biomechanical tests

At 12 weeks after surgery, a complex of muscle-Achilles tendon connected to the calcaneus was harvested (*n* = 4 for each group). Normal Achilles tendon tissue was used as a normal group. Excess tissue of the tendon-bone complex was trimmed, and the cross-sectional area (multiplying the length and width) of the repaired tendon was determined by a digital caliper. The ends of the repaired tendon that connect to the calcaneus and muscle were fixed in place using pneumatic clamps. After applying a preload of 0.1 N, complexes were subjected to uniaxial tensile tests at a rate of 5 mm/min on the Universal Testing Systems (5967, Instron, USA) until failure. If the failure does not occur in the middle of the specimen, it is need to be excluded. During testing, the forces and displacements were recorded. Stress was defined as the force divided by the initial cross-sectional area. Strain is defined as the percentage of the change in initial displacement between the clamps. The failure strain was calculated as the displacement at breakage divided by the initial displacement between the clamps. The failure load was the maximum force recorded during each test. The Young’s modulus was calculated as the slope of the stress-strain curve in the liner region beyond the initial toe region.

### Statistical analysis

Quantifications were performed from at least three independent experiments, biological replicates or sections. Data were presented as mean ± standard deviation (SD). A two-tailed Student’s *t* test was used to verify homogeneity of variances between groups. Significant analysis was performed using a one-way analysis of variance with a Tukey’s test. GraphPad Prism Software v5.0 (San Diego, California, US) was used for statistical analysis. All statistical analyses were performed with SPSS 17.0 statistical software (SPSS Inc.). All results were considered statistically significant with a *p* < 0.05.

### Reporting summary

Further information on research design is available in the Nature Research Reporting Summary linked to this article.

## Supplementary information

Revised Supplementary Information

REPORTING SUMMARY

## Data Availability

All data supporting the conclusions of this study are either provided in this published paper (and its Supplementary Information files) or available from the authors upon reasonable request.

## References

[1] Veronesi F, Torricelli P, Della Bella E, Pagani S, Fini M (2015). In vitro mutual interaction between tenocytes and adipose-derived mesenchymal stromal cells. Cytotherapy.

[2] Butler DL, Juncosa N, Dressler MR (2004). Functional efficacy of tendon repair processes. Annu. Rev. Biomed. Eng..

[3] Liu G-M (2018). Bridging repair of large rotator cuff tears using a multilayer decellularized tendon slices graft in a rabbit model. Arthrosc.: J. Arthroscopic Relat. Surg..

[4] Pan J (2014). Rotator cuff repair using a decellularized tendon slices graft: an in vivo study in a rabbit model. Knee Surg., Sports Traumatol., Arthrosc..

[5] Wong R, Alam N, McGrouther AD, Wong JKF (2015). Tendon grafts: their natural history, biology and future development. J. Hand Surg. (Eur. Vol.).

[6] Mellado JM (2005). Surgically repaired massive rotator cuff tears: MRI of tendon integrity, muscle fatty degeneration, and muscle atrophy correlated with intraoperative and clinical findings. AJR Am. J. Roentgenol..

[7] Zouani OF, Kalisky J, Ibarboure E, Durrieu MC (2013). Effect of BMP-2 from matrices of different stiffnesses for the modulation of stem cell fate. Biomaterials.

[8] Choi JS, Harley BA (2012). The combined influence of substrate elasticity and ligand density on the viability and biophysical properties of hematopoietic stem and progenitor cells. Biomaterials.

[9] Discher DE, Janmey P, Wang YL (2005). Tissue cells feel and respond to the stiffness of their substrate. Science.

[10] Qin T-W (2012). Mechanical characteristics of native tendon slices for tissue engineering scaffold. J. Biomed. Mater. Res. Part B: Appl. Biomater..

[11] Sharma RI, Snedeker JG (2012). Paracrine interactions between mesenchymal stem cells affect substrate driven differentiation toward tendon and bone phenotypes. PLoS ONE.

[12] Engler AJ, Sen S, Sweeney HL, Discher DE (2006). Matrix elasticity directs stem cell lineage specification. Cell.

[13] Ricchetti ET, Aurora A, Iannotti JP, Derwin KA (2012). Scaffold devices for rotator cuff repair. J. Shoulder Elb. Surg..

[14] Murthi AM, Ramirez MA, Parks BG, Carpenter SR (2017). Lacertus fibrosus versus Achilles allograft reconstruction for distal biceps tears: a biomechanical study. Am. J. Sports Med..

[15] Galloway MT, Lalley AL, Shearn JT (2013). The role of mechanical loading in tendon development, maintenance, injury, and repair. J. Bone Jt. Surg. Am..

[16] Qin T-W (2015). Effect of mechanical stimulation on bone marrow stromal cell–seeded tendon slice constructs: a potential engineered tendon patch for rotator cuff repair. Biomaterials.

[17] Tabata Y (2009). Biomaterial technology for tissue engineering applications. J. R. Soc. Interface.

[18] Thevenot PT (2010). The effect of incorporation of SDF-1alpha into PLGA scaffolds on stem cell recruitment and the inflammatory response. Biomaterials.

[19] Chen P (2015). Radially oriented collagen scaffold with SDF-1 promotes osteochondral repair by facilitating cell homing. Biomaterials.

[20] Kim JH, Jung Y, Kim BS, Kim SH (2013). Stem cell recruitment and angiogenesis of neuropeptide substance P coupled with self-assembling peptide nanofiber in a mouse hind limb ischemia model. Biomaterials.

[21] Klein MB, Yalamanchi N, Pham H, Longaker MT, Chang J (2002). Flexor tendon healing in vitro: effects of TGF-beta on tendon cell collagen production. J. Hand Surg. Am..

[22] Chen L, Tredget EE, Wu PY, Wu Y (2008). Paracrine factors of mesenchymal stem cells recruit macrophages and endothelial lineage cells and enhance wound healing. PLoS ONE.

[23] Hou Y (2009). The roles of TGF-beta1 gene transfer on collagen formation during Achilles tendon healing. Biochem Biophys. Res Commun..

[24] Komatsu I, Wang JH, Iwasaki K, Shimizu T, Okano T (2016). The effect of tendon stem/progenitor cell (TSC) sheet on the early tendon healing in a rat Achilles tendon injury model. Acta Biomater..

[25] Lui PP, Wong OT, Lee YW (2014). Application of tendon-derived stem cell sheet for the promotion of graft healing in anterior cruciate ligament reconstruction. Am. J. Sports Med..

[26] Ni M (2013). Engineered scaffold-free tendon tissue produced by tendon-derived stem cells. Biomaterials.

[27] Xu Y (2015). Preparation and characterization of bone marrow mesenchymal stem cell-derived extracellular matrix scaffolds. J. Biomed. Mater. Res B Appl Biomater..

[28] Rehmann MS, Luna JI, Maverakis E, Kloxin AM (2016). Tuning microenvironment modulus and biochemical composition promotes human mesenchymal stem cell tenogenic differentiation. J. Biomed. Mater. Res A.

[29] Ning LJ (2015). The utilization of decellularized tendon slices to provide an inductive microenvironment for the proliferation and tenogenic differentiation of stem cells. Biomaterials.

[30] Bi Y (2007). Identification of tendon stem/progenitor cells and the role of the extracellular matrix in their niche. Nat. Med..

[31] Yao X (2019). Stem cell extracellular matrix-modified decellularized tendon slices facilitate the migration of bone marrow mesenchymal stem cells. ACS Biomater. Sci. Eng..

[32] Li W (2019). Subcutaneously engineered autologous extracellular matrix scaffolds with aligned microchannels for enhanced tendon regeneration: Aligned microchannel scaffolds for tendon repair. Biomaterials.

[33] Wang S (2017). Decellularized tendon as a prospective scaffold for tendon repair. Mater. Sci. Eng. C. Mater. Biol. Appl..

[34] Youngstrom DW, Barrett JG, Jose RR, Kaplan DL (2013). Functional characterization of detergent-decellularized equine tendon extracellular matrix for tissue engineering applications. PLoS ONE.

[35] Yang G (2013). Enhancement of tenogenic differentiation of human adipose stem cells by tendon-derived extracellular matrix. Biomaterials.

[36] Schulze-Tanzil G, Al-Sadi O, Ertel W, Lohan A (2012). Decellularized tendon extracellular matrix—a valuable approach for tendon reconstruction?. Cells.

[37] Ning LJ (2017). Fabrication and characterization of a decellularized bovine tendon sheet for tendon reconstruction. J. Biomed. Mater. Res A.

[38] Cui, J. et al. Influence of the integrity of tendinous membrane and fascicle on biomechanical characteristics of tendon-derived scaffolds. *Biomed. Mater.* **16**, 015029 (2020). 10.1088/1748-605X/abc203 33065568

[39] Zhang CH (2018). Evaluation of decellularized bovine tendon sheets for achilles tendon defect reconstruction in a rabbit model. Am. J. Sports Med..

[40] Guo J, Chan KM, Zhang JF, Li G (2016). Tendon-derived stem cells undergo spontaneous tenogenic differentiation. Exp. Cell Res..

[41] Shen W (2012). Allogenous tendon stem/progenitor cells in silk scaffold for functional shoulder repair. Cell Transpl..

[42] Urbanczyk M, Layland SL, Schenke-Layland K (2020). The role of extracellular matrix in biomechanics and its impact on bioengineering of cells and 3D tissues. Matrix Biol..

[43] Frontera WR (2017). Physiologic changes of the musculoskeletal system with aging: a brief review. Phys. Med Rehabil. Clin. N. Am..

[44] Hsu JE, Horneff JG, Gee AO (2016). Immobilization after rotator cuff repair: what evidence do we have now?. Orthop. Clin. North Am..

[45] Yang S (2019). Oriented collagen fiber membranes formed through counter-rotating extrusion and their application in tendon regeneration. Biomaterials.

[46] Liu Y, Ramanath HS, Wang DA (2008). Tendon tissue engineering using scaffold enhancing strategies. Trends Biotechnol..

[47] He F, Chen X, Pei MJTEPA (2009). Reconstruction of an in vitro tissue-specific microenvironment to rejuvenate synovium-derived stem cells for cartilage tissue engineering. Tissue Eng. Part A.

[48] Pakyari M, Farrokhi A, Maharlooei MK, Ghahary A (2013). Critical role of transforming growth factor beta in different phases of wound healing. Adv. Wound Care (N. Rochelle).

[49] Behm B, Babilas P, Landthaler M, Schreml S (2012). Cytokines, chemokines and growth factors in wound healing. J. Eur. Acad. Dermatol. Venereol..

[50] Duperret EK, Natale CA, Monteleon C, Dahal A, Ridky TW (2016). The integrin alphav-TGFbeta signaling axis is necessary for epidermal proliferation during cutaneous wound healing. Cell Cycle.

[51] Abrahamsson SOJJOOR (1997). Similar effects of recombinant human insulin-like growth factor-I and II on cellular activities in flexor tendons of young rabbits: experimental studies in vitro. J. Orthop. Res..

[52] Shimode K (2009). Local upregulation of stromal cell-derived factor-1 after ligament injuries enhances homing rate of bone marrow stromal cells in rats. Tissue Eng. Part A.

[53] Peng R, Yao X, Ding J (2011). Effect of cell anisotropy on differentiation of stem cells on micropatterned surfaces through the controlled single cell adhesion. Biomaterials.

[54] Julier Z, Park AJ, Briquez PS, Martino MM (2017). Promoting tissue regeneration by modulating the immune system. Acta Biomater..

[55] Miller FD, Kaplan DR (2012). Mobilizing endogenous stem cells for repair and regeneration: are we there yet?. Cell Stem Cell.

[56] Yu H (2020). Bone marrow mesenchymal stem cell-derived exosomes promote tendon regeneration by facilitating the proliferation and migration of endogenous tendon stem/progenitor cells. Acta Biomater..

[57] Wang Y (2017). Stromal cell-derived factor-1 accelerates cartilage defect repairing by recruiting bone marrow mesenchymal stem cells and promoting chondrogenic differentiation. Tissue Eng. Part A.

[58] Veronesi F (2017). Mesenchymal stem cells for tendon healing: what is on the horizon?. J. Tissue Eng. Regen. Med..

[59] Dekoninck S, Blanpain C (2019). Stem cell dynamics, migration and plasticity during wound healing. Nat. Cell Biol..

[60] Lin J (2018). Cell-material interactions in tendon tissue engineering. Acta Biomater..

[61] Spiller KL, Koh TJ (2017). Macrophage-based therapeutic strategies in regenerative medicine. Adv. Drug Deliv. Rev..

[62] Morris AH, Stamer DK, Kyriakides TR (2017). The host response to naturally-derived extracellular matrix biomaterials. Semin Immunol..

[63] Sicari BM (2014). The promotion of a constructive macrophage phenotype by solubilized extracellular matrix. Biomaterials.

[64] Savitri C, Ha SS, Liao E, Du P, Park K (2020). Extracellular matrices derived from different cell sources and their effect on macrophage behavior and wound healing. J. Mater. Chem. B.

[65] Mantovani A (2004). The chemokine system in diverse forms of macrophage activation and polarization. Trends Immunol..

[66] Spiller KL (2015). Sequential delivery of immunomodulatory cytokines to facilitate the M1-to-M2 transition of macrophages and enhance vascularization of bone scaffolds. Biomaterials.

[67] Zhu M (2019). In vivo engineered extracellular matrix scaffolds with instructive niches for oriented tissue regeneration. Nat. Commun..

[68] Sharma P, Maffulli N (2005). Tendon injury and tendinopathy: healing and repair. J. Bone Joint. Surg. Am..

[69] Melincovici CS (2018). Vascular endothelial growth factor (VEGF) - key factor in normal and pathological angiogenesis. Rom. J. Morphol. Embryol..

[70] Petersen W (2003). The angiogenic peptide vascular endothelial growth factor (VEGF) is expressed during the remodeling of free tendon grafts in sheep. Arch. Orthop. Trauma Surg..

[71] Molloy T, Yao W, Murrell GJSM (2003). The roles of growth factors in tendon and ligament healing. Sports Med..

[72] Yang B (2010). Development of a porcine bladder acellular matrix with well-preserved extracellular bioactive factors for tissue engineering. Tissue Eng. Part C. Methods.

[73] Murrell GAC (1992). The achilles functional index. J. Orthop. Res..

