# Supplementary material for: Biomechanically and biochemically functional scaffold for recruitment of endogenous stem cells to promote tendon regeneration
Source: NPJ Regen Med. 2022 Apr 26;7:26. doi: 10.1038/s41536-022-00220-z (PMC9043181; doi:10.1038/s41536-022-00220-z)
Supplement: Supplementary file 1 — Revised Supplementary Information [file 41536_2022_220_MOESM1_ESM.pdf]

Biomechanically and biochemically functional scaffold for recruitment of endogenous stem cells to promote tendon regeneration

Jing Cui [1], Liang-Ju Ning [1], Fei-Peng Wu [2, 3], Ruo-Nan Hu [1], Xuan Li [1], Shu-Kun He [2], Yan-Jing Zhang [1], Jia-Jiao Luo [1], Jing-Cong Luo [1], Ting-Wu Qin [1,*]

[1] Laboratory of Stem Cell and Tissue Engineering, Orthopedic Research Institute, State Key Laboratory of Biotherapy and Cancer Center, West China Hospital, Sichuan University and Collaborative Innovation Center of Biotherapy, Chengdu, China

[2]Department of Orthopedic Surgery, West China Hospital, Sichuan University, Chengdu, China

[3]Panzhihua Central Hospital, Panzhihua, China

***Corresponding Author:**

Ting-Wu Qin, Ph.D.

Lab of Stem Cell and Tissue Engineering,

State Key Laboratory of Biotherapy,

West China Hospital,

Sichuan University,

Chengdu, Sichuan 610041, P.R. China

Tel:+86-28-85164090; Fax:+86-28-85164088

E-mail: tingwuqin@hotmail.com

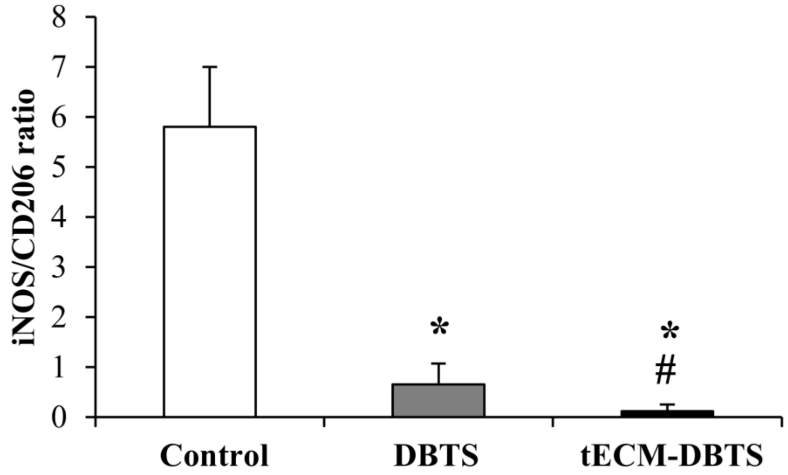

Supplementary Figure 1. Quantification of M1-type (iNOS) / M2-type (CD206) macrophages ratio in the wounds in the rat model (n=5). * signifies a *p* value of < 0.05 as compared to the control. # signifies a *p* value of < 0.05 as compared to the DBTS.

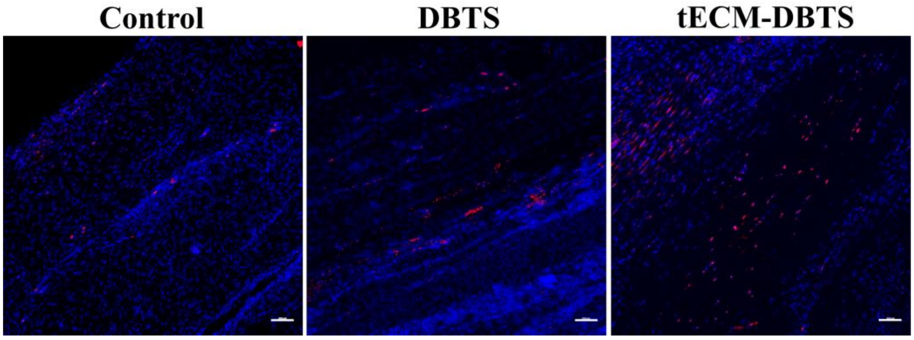

Supplementary Figure 2. Immunofluorescence staining of TNMD (red) of the control, DBTS and tECM-DBTS at 2 weeks. Scale bars, 100 μm.

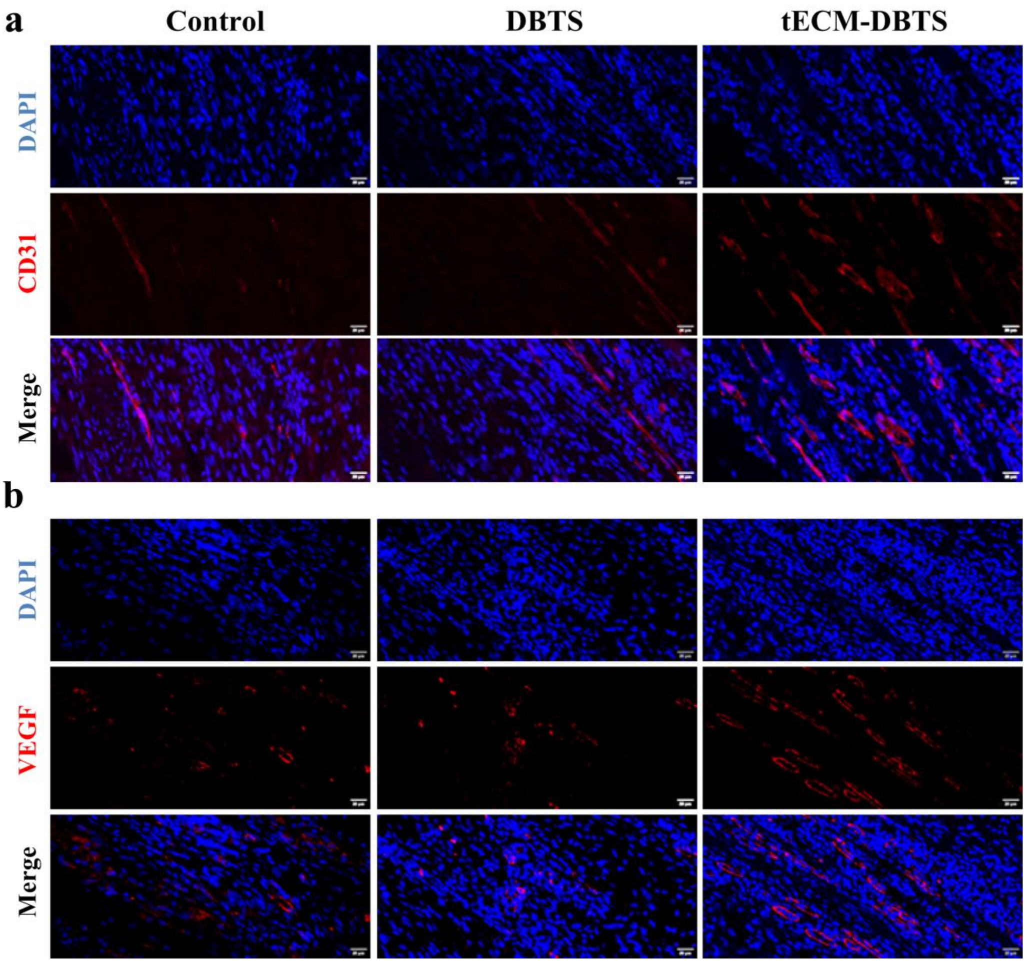

Supplementary Figure 3. Immunofluorescence staining of CD31 (red) and VEGF (red) of the control, DBTS and tECM-DBTS at 4 weeks. Scale bars, 20 μm.

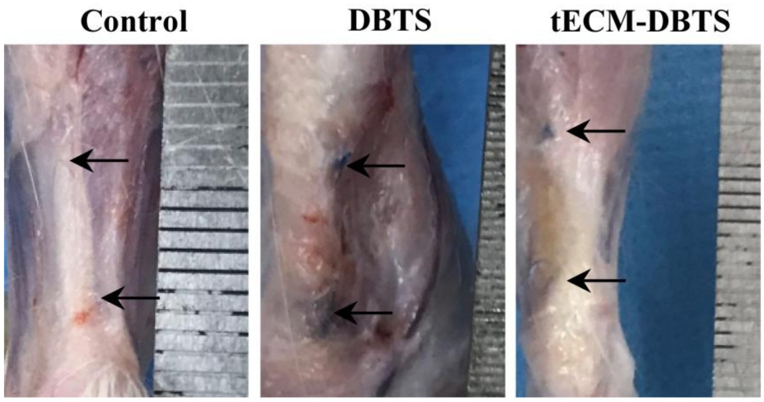

Supplementary Figure 4. Representative images of the gross morphology of the remodeled control, DBTS and tECM-DBTS at 12 weeks. Black arrows indicate the suture sites.

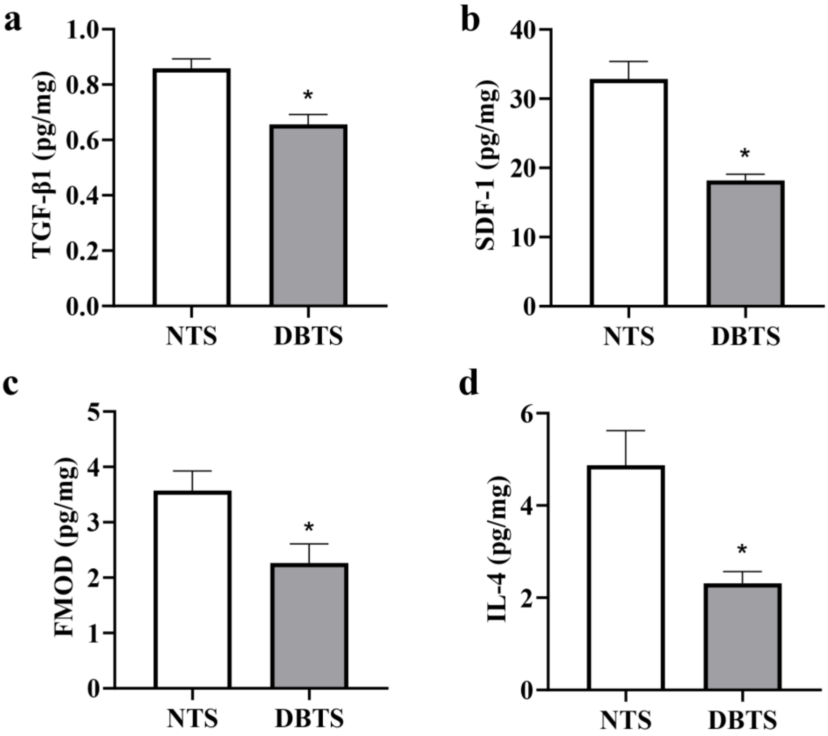

Supplementary Figure 5. The contents of TGF-β1 (a), SDF-1(b), FMOD (c) and IL-4 (d) in the NTS and DBTS scaffolds were determined by ELISA analysis (n=3). * signifies a *p* value of < 0.05 as compared to the NTS.
