# Supplementary material for: Biomechanically and biochemically functional scaffold for recruitment of endogenous stem cells to promote tendon regeneration
Source: NPJ Regen Med. 2022 Apr 26;7:26. doi: 10.1038/s41536-022-00220-z (PMC9043181; doi:10.1038/s41536-022-00220-z)
Supplement: Supplementary file 2 — REPORTING SUMMARY [file 41536_2022_220_MOESM2_ESM.pdf]

nature portfolio

Corresponding author(s): Professor Ting-wu Qin

Last updated by author(s): Mar 6, 2022

Reporting Summary

Nature Portfolio wishes to improve the reproducibility of the work that we publish. This form provides structure for consistency and transparency in reporting. For further information on Nature Portfolio policies, see our Editorial Policies and the Editorial Policy Checklist.

Statistics

For all statistical analyses, confirm that the following items are present in the figure legend, table legend, main text, or Methods section.

n/a	Confirmed	
☐	☒	The exact sample size (n) for each experimental group/condition, given as a discrete number and unit of measurement
☐	☒	A statement on whether measurements were taken from distinct samples or whether the same sample was measured repeatedly
☐	☒	The statistical test(s) used AND whether they are one- or two-sided<br>*Only common tests should be described solely by name; describe more complex techniques in the Methods section.*
☐	☒	A description of all covariates tested
☐	☒	A description of any assumptions or corrections, such as tests of normality and adjustment for multiple comparisons
☐	☒	A full description of the statistical parameters including central tendency (e.g. means) or other basic estimates (e.g. regression coefficient) AND variation (e.g. standard deviation) or associated estimates of uncertainty (e.g. confidence intervals)
☐	☒	For null hypothesis testing, the test statistic (e.g. F, t, r) with confidence intervals, effect sizes, degrees of freedom and P value noted<br>*Give P values as exact values whenever suitable.*
☐	☒	For Bayesian analysis, information on the choice of priors and Markov chain Monte Carlo settings
☐	☒	For hierarchical and complex designs, identification of the appropriate level for tests and full reporting of outcomes
☐	☒	Estimates of effect sizes (e.g. Cohen's d, Pearson's r), indicating how they were calculated

Our web collection on statistics for biologists contains articles on many of the points above.

Software and code

Policy information about availability of computer code

Data collection	No software was used.
Data analysis	No software was used.

For manuscripts utilizing custom algorithms or software that are central to the research but not yet described in published literature, software must be made available to editors and reviewers. We strongly encourage code deposition in a community repository (e.g. GitHub). See the Nature Portfolio guidelines for submitting code & software for further information.

Data

Policy information about availability of data

All manuscripts must include a data availability statement. This statement should provide the following information, where applicable:

- Accession codes, unique identifiers, or web links for publicly available datasets
- A description of any restrictions on data availability
- For clinical datasets or third party data, please ensure that the statement adheres to our policy

All data supporting the conclusions of this study are either provided in this published paper (and its Supplementary Information files) or available from the authors upon reasonable request.

Field-specific reporting

Please select the one below that is the best fit for your research. If you are not sure, read the appropriate sections before making your selection.

☒ Life sciences ☐ Behavioural & social sciences ☐ Ecological, evolutionary & environmental sciences

For a reference copy of the document with all sections, see nature.com/documents/nr-reporting-summary-flat.pdf

Life sciences study design

All studies must disclose on these points even when the disclosure is negative.

Sample size	n = 3-5 samples per group.
Data exclusions	The data from failure not at the middle of specimens and misoperation need to be excluded.
Replication	yes.
Randomization	yes.
Blinding	yes.

Reporting for specific materials, systems and methods

We require information from authors about some types of materials, experimental systems and methods used in many studies. Here, indicate whether each material, system or method listed is relevant to your study. If you are not sure if a list item applies to your research, read the appropriate section before selecting a response.

Materials & experimental systems

n/a	Involved in the study	
☐	☒	Antibodies
☒	☐	Eukaryotic cell lines
☒	☐	Palaeontology and archaeology
☐	☒	Animals and other organisms
☒	☐	Human research participants
☒	☐	Clinical data
☒	☐	Dual use research of concern

Methods

n/a	Involved in the study	
☒	☐	ChIP-seq
☒	☐	Flow cytometry
☒	☐	MRI-based neuroimaging

Antibodies

Antibodies used	Anti-CD68 antibody (1:100; ab31630); Anti-mannose receptor anti-CD206 (1:20000; ab64693); Anti-iNOS antibody (1:400; ab15323); Anti-CD146 (1:200; ab75769); Anti-CD44 (1:200; ab157107); Anti-TNMD antibody (1:200; bs-7525R); Anti-CD31 antibody (1:100; ab28364); Anti-VEGF antibody (1:200; YM3681).
Validation	Validated with rats.

Animals and other organisms

Policy information about studies involving animals; ARRIVE guidelines recommended for reporting animal research

Laboratory animals	Sixty male Sprague-Dawley rats.
Wild animals	The study did not involve wild animals.
Field-collected samples	The animals were housed in ventilated cages with a light / dark cycle of 12:12 hours and free access to food and water. At the preset time points, the experimental samples were obtained in the animal operating room, which was well ventilated and pathogen-free.
Ethics oversight	The Animal Ethics Committee of West China Hospital of Sichuan University (NO. 2019293A).

Note that full information on the approval of the study protocol must also be provided in the manuscript.
